# Extracting Teeth under the Influence of Anæsthetics in the Lying-Down Posture

**Published:** 1872-09

**Authors:** E. M. Morrison

**Affiliations:** Noblesville, Ind.


### Extracting Teeth Under the Influence of Anæsthetics in the Lying-Down Posture.

BY E. M. MORRISON, M. D., NOBLESVILLE, IND.

*Read Before the Indiana State Dental Association, June 26th,* 1872.

The practability of this manner of operating has for some time past engaged my attention, and from various experiments and trials in this direction, my conclusions are favorable; the results are gratifying. It is universally conceded by good authority that the erect sitting or reclining posture is very unfavorable and dangerous in the administration of anæsthetics; but it has been claimed on various grounds, well-known to most of us, that the lying-down posture was not practicable in the extraction of teeth and other operations in the mouth. On account of this alleged *impracticability* most dentists operate in their chairs, in the sitting or reclining posture. In fact, so far as I know, all do this, but we are sometimes forced to give up old theories and practices for new lights—truths new to us; and change the manner of our operations for safety to our patients, or for durability and efficiency, even conceding them in some cases to be more inconvenient to us.

As intimated already, I have become fully satisfied that the extraction of teeth and other surgical operations in the mouth are practicable in the lying-down posture without very great difficulty. For a long time I have refused to administer chloroform or any other anæsthetic by myself, and on this account, Drs. J. M. Gray and W. B. Graham have each frequently been called into my office for the purpose of administering chloroform. In these cases the operations have mostly been performed in the reclining posture for want of suitable apparatus; but the experiments and observations have convinced all of us of the practicability of such operations on the patient when lying down. Dr. Gray even spoke to me on the subject before I had mentioned the object of my experiments to him.

To insure success the office should be supplied with a high operating table, with suitable cushions and pillows—a good lounge might answer—but the hight of either should be easily adjusted to suit the convenience of the operator. The patient should receive the usual preliminary examination of course, to see that the conditions of the system are favorable, and when ready be placed upon the table, as for other important surgical operations. When every thing else is in readiness the patient should be brought thoroughly under the influence of the anæsthetic before commencing the operation. In this way complete control over the patient is attained, and in my opinion the danger lessened. Plenty of water and small fine sponges are necessary, and should be at hand; and as soon as a tooth is extracted your assistant must be able to apply a sponge, squeezed out of the water, so dextrously and firmly to the gum and alveolus as to prevent the escape of blood, and not cover up the next tooth to be extracted. As fast as the teeth are extracted the sponge should be extended over the gum or new ones applied. In this way a goodly number of teeth may be taken out without a flow of blood sufficient to interfere with the progress of the operation, or endanger the functions of respiration, which is especially to be guarded. Of course a reasonable amount of manipulative skill is required in these operations, but for these I have felt no lack; on the other hand I have been blessed with suggestive and co-operative skill of great value. If the escape of blood should accidently be considerable, the patient's head can be turned to one side, and the mouth freed from the excess as easily as if sitting up on a chair. If proper skill has been exercised, however, this will not often be necessary before the patient is partially restored to consciousness, at least, and it may not be necessary at all. A little practice will be necessary, and a few partial failures will most likely occur before the results are satisfactory, especially as the proper application of the sponges requires the greatest amount of skill in execution. In view of the facts which I have cited, and the additional fact that *emotional syncope* is a frequent cause of death, every dentist should be prepared with a ready means of placing his patients in a lying-down posture. Happily our floors are level, and patients can be quickly, if not easily, taken from our chairs in cases of danger, and laid down on the floor. We should try to devise some more elegant and efficient means, however, of averting these accidents, and the appalling calamities so frequently occurring in the hands of our profession from these causes. Whether we are guilty of any mismanagement or not the charge will rest upon us, and the public mind is not easily disabused, yet the lookout is getting more hopeful. Some of the old ruts that we have traveled in for the last quarter of a century or more have recently been filled up as it were; new lights have shown upon us, and we have been elevated to a higher standard. I hope to the true level in some things. Be this as it may, old notions have vanquished from our minds under the co-operative labors of our profession, and we have almost unconsciously advanced in the line of substantial improvement; and while we have thus advanced we have as surely grown in public favor. *The more pains-taking we are, the better we are appreciated.* Then I hope the lying-down posture will receive a fair share of attention and an impartial trial in cases where anæsthetics are employed. It will be a pains-taking that will add greatly to the safety of our patients, perhaps save many lives, and elevate us in public favor, and make our services better appreciated.

I find some comments in the *London Lancet*, of May 25th on the death of Mrs. O'Shaughnessy, who died in Dr. Newbrough's office in New York, on the 20th of March last; so well-timed that I will close this article with a reproduction of a few of them:

"A jury of ten persons, each of whom wrote M. D. after his name, rendered an elaborate verdict commencing with the finding that the deceased "came to her death from asphyxia or apnœa, as evidenced by the symptoms manifested by the patient before death and the conditions found at the *post mortem*; the asphyxia having in our opinion been induced by the inhalation of gas administered."

After reviewing the testimony, which I can not now lay before you, the paper further says: "Upon this testimony, however, the facts seem to admit of only one conclusion. The nitrous oxide could have had no more to do with the fatal issue, either directly or indirectly, than if it had never been brought into the room. The patient manifestly fainted from terror, doing so as soon as her state of mental tension was relaxed by the operation being completed. Her syncope was just a result of the reaction of an overstrung nervous system; and, if Mr. Newbrough had only laid her flat on the floor, she would probably have recovered in five minutes. * * * It was the unwisely holding her upright that determined the fatal issue; and although one must sympathize with the queer medical jury to the extent of admitting that no anæsthetic agent should be entrusted to a man who treats syncope by the erect posture, yet still, when we consider the wide prevalence of his error, we must not be too hard on the dentist. * * * * Moreover, "sitting up" is the most common cause of death after enfeebling illness."

Instances are cited in the paper where death has occurred from sitting up, or being held up in syncope, and from this and other causes just before the anticipated administration of anæsthetics, in which a possible coincidence might have easily placed the blame on the anæsthetic. It concludes as follows: "The absence in Mrs. O'Shaughnessy's case of any apparent cause of sudden death other than emotional syncope and the upright posture, and the entire sufficiency of these to explain the occurrence, seem to render the verdict of the New York jury one of the most astounding on record. It can hardly fail to be challenged by the American medical journals, and we shall be curious to see what attempts are made to justify it."

